# Ambient particulate matter enhances the pulmonary allergic immune response to house dust mite in a BALB/c mouse model by augmenting Th2‐ and Th17‐immune responses

**DOI:** 10.14814/phy2.13827

**Published:** 2018-09-19

**Authors:** Alejandro R. Castañeda, Christoph F. A. Vogel, Keith J. Bein, Heather K. Hughes, Suzette Smiley‐Jewell, Kent E. Pinkerton

**Affiliations:** ^1^ Center for Health and the Environment University of California Davis California; ^2^ Department of Environmental Toxicology University of California Davis California; ^3^ Air Quality Research Center University of California Davis California; ^4^ M.I.N.D. Institute University of California Davis California; ^5^ Department of Pediatrics School of Medicine University of California Davis California

**Keywords:** Allergic airway inflammation, allergy, house dust mite allergen, particulate matter, Th17 immunity, Th2 immunity

## Abstract

Ambient particulate matter (PM) exacerbates airway inflammation and hyper‐reactivity in asthmatic patients. Studies show that PM has adjuvant‐like properties that enhance the allergic inflammatory response; however, the mechanisms through which PM enhances these processes remain elusive. The objective of the study was to examine how ambient PM enhances the allergic immune response. Eight‐week‐old BALB/c mice were sensitized with house dust mite (HDM) or HDM and ambient particulate matter (PM, 2.5 *μ*m; Sacramento, CA) to assess how PM modulates the development of adaptive immune responses against allergens. Both groups were challenged with HDM only. Bronchoalveolar lavage (BAL) was analyzed for extent of airway inflammation. Lung tissue was used for histological analysis, mucosubstance quantification, and heme oxygenase‐1 (HO‐1) localization/quantification. Gene expression was analyzed in whole lung to characterize immune markers of inflammation: cytokines, chemokines, antioxidant enzymes, and transcription factors. Cytokine and chemokine protein levels were quantified in whole lung to confirm gene expression patterns. Compared to HDM‐only sensitization, exposure to PM during HDM sensitization led to significant immune cell recruitment into the airway subepithelium, IgE gene expression, mucosubstance production, and Th2‐associated cytokine expression. HO‐1 levels were not significantly different between the treatment groups. Gene expression profiles suggest that polycyclic aromatic hydrocarbon (PAH) content in PM activated the aryl hydrocarbon receptor (AhR) and enhanced Th17‐responses in the mice that received HDM and PM compared to mice that received HDM‐only. The findings suggest that PM enhances allergic sensitization via enhancement of Th2‐mediated inflammation and that AhR activation by PAHs in PM promotes Th17‐immune responses.

## Introduction

In the last two decades the incidence of asthma in the California's Central Valley has risen dramatically. In fact, 16 of the 17 counties in the Central Valley have higher rates of active asthma (county rates range from 7.8% to 14.7%) for all age groups than the national average (7.7%) (Centers for Disease Control and Prevention, 2014; California Breathing, 2016). The Central Valley also has one of the most severe air quality problems in the United States with respect to PM pollution (Herner et al. 2005). Since 2003, the Central Valley has continuously been designated a non‐attainment area for both PM_2.5_ (defined as exceeding an annual mean of 12 *μ*g/m^3^ concentration of PM_2.5_) and PM_10_ (defined as exceeding either an annual mean of 20 *μ*g/m^3^ concentration of PM_10_, or a 24 h average of 50 *μ*g/m^3^ concentration of PM_10_) by the state of California regulatory guidelines, which are more stringent than federal guidelines (California Air Resource Board, 2016; Environmental Protection Agency, 2016).

Numerous studies have demonstrated that exposure to PM facilitates allergen sensitization and worsens asthmatic symptoms in children (Mortimer et al. 2008; Fuertes et al. 2013; Bowatte et al. 2015; Fuertes and Heinrich 2015). For example, living in close proximity to major highways has been strongly associated with a higher risk of asthma in children with no family history of atopy (McConnell et al. 2006), a fact that has been attributed to chronic exposure to PM byproducts of gasoline and diesel engines. We previously reported that mice exposed to PM2.5 during ovalbumin sensitization produced enhanced allergic airway inflammation upon subsequent allergen challenge in the absence of PM, relative to mice sensitized and challenged with allergen‐only (Castaneda et al. 2017). This suggests that PM has long‐term effects in its ability to modulate the immune system, possibly by affecting the development of the adaptive immune response. However, the mechanisms by which PM mediated these effects were unclear.

Our model showed evidence of enhanced oxidative stress via heme oxygenase‐1 (HO‐1) protein levels. This finding is in agreement with other investigators who postulate that PM enhances disease via oxidative stress (Ayres et al. 2008). Yet, our results suggested that oxidative stress was formed through endogenous processes rather than exogenous PM‐mediated processes as mice were exposed to PM only during sensitization, allowing sufficient time for PM to be cleared by the time mice were challenged with allergen‐only and inflammation was assessed (10 days later).

The purpose of this study was to investigate whether exposure to PM2.5 during allergen sensitization enhances the classical Th2‐immune response associated with allergy or whether it simultaneously promotes other immune responses (i.e., Th1‐, Th17‐, or T regulatory immune responses) alongside the Th2‐immune response. Understanding how PM modulates the immune response to exacerbate allergy will provide an important step in elucidating such PM‐mediated mechanisms in future studies. The study presented here was performed using the same experimental design from our preceding study and PM source/size, with the exception that the house dust mite (HDM) allergen, a human allergen, was used rather than ovalbumin. Briefly, young BALB/c mice were sensitized with HDM via intranasal instillation on days 1, 3 and 5 followed by allergen challenge on days 12–14 to elicit an allergic inflammatory response. Atmospheric PM2.5 collected from the city of Sacramento, located in California's Central Valley, was administered intranasally, with or without HDM, only during allergic sensitization.

To assess how PM modulates the immunological response to allergens, we assessed various immunological endpoints after allergen challenge: bronchoalveolar (BAL) cellular profiles, lung histopathology, gene expression patterns of cytokines, chemokines, dendritic cell activation markers, and notably adaptive immune responses (Th1, Th2, Th17, or Treg immune responses). These endpoints were used to gain insights into mechanisms by which PM2.5 shapes the development of the immune response, ultimately leading to exacerbation of the allergic/adaptive immune response.

## Methods and Materials

### Ambient PM collection, extraction, and chemical characterization

Ambient PM was collected in the summer of 2011 at an urban sampling site located on the rooftop of a two‐story building at the northeast corner of T St. and 13th St. in downtown Sacramento, CA. The sampling site is surrounded by a mixture of residential, commercial and industrial sources and within a quarter mile of a major freeway interchange. Chemical characterization was performed to identify trace metals by inductively coupled ion mass spectroscopy (IC‐MS), water soluble inorganic and organic ions by ion chromatography and atomic absorption spectrophotometry (AAS), molecular organic compounds by thermal desorption gas chromatography‐mass spectroscopy (GC‐MS) and elemental and organic carbon by thermal optical reflectance. Lipopolysaccharide (LPS) levels were quantified by the Lonza Kinetic Chromogenic LAL Endotoxin Assay (Basel, Switzerland).

### Animal model: allergen and particulate matter administration

Eight‐week‐old male BALB/c mice were obtained from Harlan Laboratories (Livermore, CA). Animals were housed at the University of California, Davis (UC Davis), Center for Health and the Environment. Experiments were approved by the UC Davis Institutional Animal Care and Use Committee.

**Figure 1:**
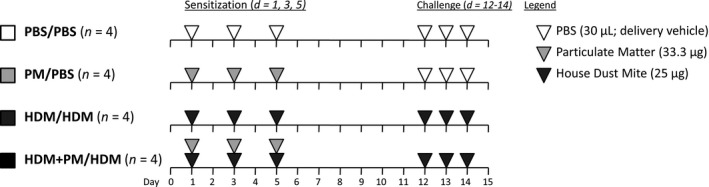
Allergic sensitization and challenge protocol. BALB/c mice were sensitized (day 1, 3, and 5) and challenged (day 12–14) intranasally with either PBS (30 *μ*L/day; delivery vehicle; white triangles; *n* = 4), PM (33.3 *μ*g/day, light grey triangles; *n* = 4), HDM (25 *μ*g/day, dark grey triangles; *n* = 4) or HDM + PM (*n* = 4). Mice were euthanized 24 h after the final challenge (day 15) to assess pulmonary inflammation.

Mice were acclimated for 2 weeks and then randomly divided into four groups that were treated to the sensitization and challenge protocol illustrated in Figure 1. HDM allergen sensitization was performed on days 1, 3, and 5. HDM allergen challenge was performed on days 12–14. The four groups were sensitized/challenged as follows: (1) PBS/PBS (2) PM/PBS, (3) HDM/HDM, or (4) HDM + PM/HDM.

PM and HDM were administered in a total volume of 30 *μ*L/day/mouse of PBS (delivery vehicle) via intranasal instillation. HDM (Greer Laboratories, Lenoir, NC) was administered at a dose of 25 *μ*g/day/mouse; a 25 *μ*g dose of HDM contained 161.8 endotoxin units. PM was sonicated for 15 min prior to administration, and a dose of 33.3 *μ*g/day/mouse (100 *μ*g total sensitization dose) was used. PM was only administered during the sensitization period in both PM/PBS and HDM + PM/HDM groups to assess its adjuvant‐like effect on the adaptive response (challenge). In mice sensitized to HDM + PM, HDM, and PM were dosed separately, approximately 15 min apart, to avoid particle‐protein interactions. For detailed information regarding this experimental protocol, we refer the reader to the literature (Castaneda and Pinkerton 2016). Animals were euthanized on day 15, 24‐h after the final intranasal challenge, to assess pulmonary inflammation.

### Bronchoalveolar lavage and cellular analysis

Mice were intratracheally cannulated, and the left lung was clamped. The right lung was lavaged with two volumes of 0.3 mL of cold sterile PBS (Sigma Aldrich, St. Louis, MO). The lavage fluid was centrifuged at 500*g* for 15 min at 4°C. The supernatant was removed and frozen, while cells were resuspended in 500 *μ*L of PBS. Cell numbers and viability was determined via hemocytometer using 0.4% trypan blue solution (Sigma‐Aldrich). Cells were centrifuged onto slides (1.5 × 10^3^ cells/slide) using a Shandon Cytospin (Thermo Shandon, Inc., Pittsburg, PA). Slides were stained with hematoxylin and eosin (H&E; American MasterTech, Lodi, CA) for cellular differential analysis. A total of 500 cells were counted per slide to determine macrophage, neutrophil, eosinophil, and lymphocyte cell composition of bronchoalveolar lavage.

### Histological analysis

The non‐lavaged left lung lobe was collected and inflation fixed with 4% paraformaldehyde (Electron Microscopy Sciences, Hatfield, PA) at 30 cm water pressure for one hour. Fixed lungs were embedded in paraffin wax and sectioned into 5 *μ*m sections that were mounted onto slides. Slides were stained with H&E, combined eosinophil and mast cell (CEM) stain, alcian blue and periodic acid Schiff (ABPAS), or used for immunohistochemistry.

### Blood plasma collection and immunoglobulin analysis

Blood was collected immediately following euthanasia via cardiac puncture in EDTA coated cryotubes (BD, Franklin Lakes, NJ) and centrifuged at 3000*g* for 10 min at 4°C to collect plasma. Plasma was used to quantify total immunoglobulin E (IgE) levels via ELISA (Biolegend, San Deigo, CA) according to the manufacturer's instructions.

### ABPAS/mucosubstance morphometric analysis

Intraepithelial mucosubstance production was quantified along the main axial‐path airway at four different levels in the left lung lobe: Level 1 corresponds to the main apical‐path airway whereas Levels 2, 3, and 4 correspond to airway generations 2–3, 5, and 10, respectively, along the main axial‐path airway. At each level, the airway was divided into quadrants. One image of the airway epithelium was captured at 400× magnification in each quadrant and used for morphometric analysis using ImageJ (National Institutes of Health, Bethesda, MD). A Mertz grid (rolling cycloid arc grid) with 518 points per image was overlaid on the captured image. Points that intersected mucosubstances, basal lamina, and epithelium were counted to calculate the volume of intraepithelial mucosubstances over the area of the basal lamina.

### Immunohistochemistry

Staining of lung tissue sections for HO‐1 was performed using EDTA (Sigma Aldrich; 1 mmol/L, pH 8.0) antigen retrieval for 2 min at 123°C, 18 psi. Endogenous peroxidase activity was blocked with 3% hydrogen peroxide (Sigma Aldrich) for 10 min. Tissue sections were treated with protein block (Dako, Carpinteria, CA) for 10 min followed by binding of primary HO‐1 IgG antibody (Abcam, Cambridge, MA; ab13243, anti‐mouse made in rabbit) at a dilution of 1:400 (2.5 *μ*g/mL) or for 1 h. Primary antibody was linked to EnVision System HRP‐labeled anti‐rabbit polymer (Dako) for 30 min then treated with DAB substrate chromogen system (Dako) for 5 min. Tissue sections were counterstained with hematoxylin and coverslipped. A negative control consisted of non‐immune IgG substituted for the primary HO‐1 antibody.

### Gene expression analysis

RNA was preserved from the right lung caudal lobe using TRI Reagent^®^ (Sigma‐Aldrich) and extracted with an RNA isolation kit (Zymo Research, Irvine, CA). RNA was converted to cDNA using Applied Biosystem's (Indianapolis, IN) High‐Capacity cDNA Reverse Transcription Kit. Gene‐specific mouse primers, cDNA (1 *μ*g/reaction) and SYBR Green (Applied Biosystem) were used for quantitative PCR (qPCR). Gene expression was assessed using the ΔΔ‐Ct method and standardized to the expression of GAPDH or EEF1A1 housekeeping genes. Mouse gene primers were designed using Primer3 primer design software(Untergasser et al. 2012). The primer sequences used in this study are listed in Table 1.

**Table 1:** Primer sets for gene expression analysis

Gene	Forward (5′ → 3′)	Reverse (5′ → 3′)
IL‐1*β*	GGGCCTCAAAGGAAAGAATC	TACCAGTTGGGGAACTCTGC
IL‐4	TCAACCCCCAGCTAGTTGTC	TGTTCTTCGTTGCTGTGAGG
IL‐5	GAAGTGTGGCGAGGAGAGAC	GCACAGTTTTGTGGGGTTTT
IL‐6	AGTTGCCTTCTTGGGACTGA	TCCACGATTTCCCAGAGAAC
IL‐13	CAGCTCCCTGGTTCTCTCAC	CCACACTCCATACCATGCTG
IL‐17A	AGGCCCTCAGACTACCTCAACCGTTCC	TGGTCCAGCTTTCCCTCCGCATT
IL‐22	TTTCCTGACCAAACTCAGCA	TCTGGATGTTCTGGTCGTCA
IL‐25	ATGGGAAGAAGCTGATGGTG	TAGAGCCAGCTGGTTGTCCT
IL‐33	ATGGGAAGAAGCTGATGGTG	TAGAGCCAGCTGGTTGTCCT
TNF*α*	AGCCCCCAGTCTGTATCCTT	CTCCCTTTGCAGAACTCAGG
CCL1	GGATGTTGACAGCAAGAGCA	TAGTTGAGGCGCAGCTTTCT
CCL3	AGATTCCACGCCAATTCATC	CTCAAGCCCCTGCTCTACAC
CCL4	CCCACTTCCTGCTGTTTCTC	GAGGAGGCCTCTCCTGAAGT
CXCL2	AAGTTTGCCTTGACCCTGAA	AGGCACATCAGGTACGATCC
CXCL5	GAAAGCTAAGCGGAATGCAC	GGGACAATGGTTTCCCTTTT
DUOX1	CTGGACATCCTGGTGGTCTT	CAGGTCAGCTCCTCCTTGTC
GPX1	GAGGGTAGAGGCCGGATAAG	AGAAGGCATACACGGTGGAC
PRDX1	TTTTGGGCAGACCAATCTTC	CATGCTGGGGAAACATTCTT
PRDX3	TGGACACCAGAGTCCCCTAC	TCAAGGCATTGGAAGGATTC
SOD3	ATCCCACAAGCCCCTAGTCT	GTGCTATGGGGACAGGAAGA
CD80	TTCGTCTTTCACAAGTGTCTTCA	TGCCAGTAGATTCGGTCTTCA
CD83	TCTGGACGAGTCACTTGTGG	TATGACAGGCATTCGCTCAG
CD86	GAAGCCGAATCAGCCTAGC	CAGCGTTACTATCCCGCTCT
MHCII	GAGTCACACCCTGGAAAGGA	ACAGCCTCAGGGTCAAGAGA
IDO1	GGCTAGAAATCTGCCTGTGC	AGAGCTCGCAGTAGGGAACA
AHR	ACCAGAACTGTGAGGGTTGG	TCTGAGGTGCCTGAACTCCT
AHRR	TATGGTAGAGGCCAGGAACC	GCTGCCTTTTTGTCCCTAAG
FOXP3	AGAAGCTGGGAGCTATGCAG	GCTACGATGCAGCAAGAGC
GATA3	GTGGTCACACTCGGATTCCT	GCAAAAAGGAGGGTTTAGGG
RORgT	GGCTTTCAGGCTTCATGG	ACTTCCATTGCTCCTGCTGT
T‐BET	CCTGGACCCAACTGTCAACT	AACTGTGTTCCCGAGGTGTC
IgE	TGATCTCAAACAGCCAGCAC	CTTCCCCACCACAGCTACAT
EEF1A1	GCATGGTGGTTACCTTTGCT	CAGCAACATTGCCTCGTCTA

### Pulmonary cytokine analysis

The right lung middle lobe was homogenized with a cell lysis kit (Bio‐Rad, Hercules, CA). Total protein concentration was assessed via Lowry protein assay (Bio‐Rad). Lung homogenates were diluted at various concentrations: 1:50, 1:100, 1:200 in 1% BSA (Sigma Aldrich) for ELISA. CCL5, CXCL1, IL‐5, IL‐6, IL‐17A, IL‐25, IL‐33, and TNF‐*α* protein levels were measured using Biolegend or R&D Systems (Minneapolis, MN) ELISA kits according to the manufacturer's protocol. Protein levels were standardized to total lung protein.

### Statistical method

Data are expressed as means ± standard error of the mean (SEM). All comparisons (PBS/PBS, PM/PBS, HDM/HDM, and HDM + PM/HDM) were assessed by one‐way ANOVA followed by post hoc Tukey's Multiple Comparison Test using GraphPad PRISM 5 software. A value of *P < *0.05 was considered statistically significant.

## Results

### PM chemical composition

The summertime PM_2.5_ collected for this study in Sacramento was dominated by organic carbon (49% composition by mass), including a myriad of polycyclic aromatic hydrocarbons (PAHs) and nonaromatic hydrocarbons, and water soluble inorganic ions (21% composition by mass). Elemental carbon accounted for 1.4% of PM mass, and various metals ranging from lithium to lead were detected at levels significantly above detection limits. Further detailed descriptions of how the PM for this study was collected, extracted, and characterized can be found in the literature (Bein and Wexler 2014, 2015; Castaneda and Pinkerton 2016; Castaneda et al. 2017). Endotoxin levels in the collected PM sample was found to be below the limit of detection (LOD) of <0.005 endotoxin units.

### Lung inflammatory response

**Figure 2:**
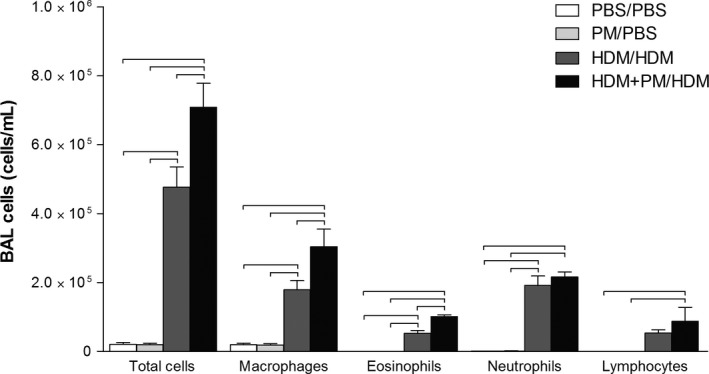
Cellular profiles of recovered bronchoalveolar lavage (BAL) fluid. PM enhanced HDM‐induced allergic airway inflammation compared to HDM treatment‐alone. BALB/c mice were sensitized/challenged with PBS/PBS(control; white), PM/PBS (light gray), HDM/HDM (dark grey), or HDM + PM/HDM (black). Total cells, macrophages, eosinophils, neutrophils, and lymphocytes are shown in number of cells counted per milliliter (cells/mL). Data are presented as mean ± SEM (*n *=* *4/group). Bars indicate a significant difference of *P *< 0.05 between groups.

PM exposure during allergic sensitization led to greater pulmonary immune cell influx upon allergen challenge (Fig. 2). Animals sensitized with HDM + PM had a significantly higher number of total BAL immune cells, macrophages, and eosinophils compared to mice sensitized with HDM in the absence of PM. The higher influx of eosinophils, which are involved in allergy and Th2 immune responses, into the lungs of HDM + PM/HDM‐treated mice compared to the HDM/HDM group, suggests that PM promotes the allergic immune response. Notably, PM/PBS treatment was comparable to the PBS/PBS control, failing to produce any changes in immune cell migration to the lung, suggesting PM acts as an immune adjuvant in modulating the immune response to the HDM allergen but does not produce discernible effects by itself.

### Histopathology

**Figure 3:**
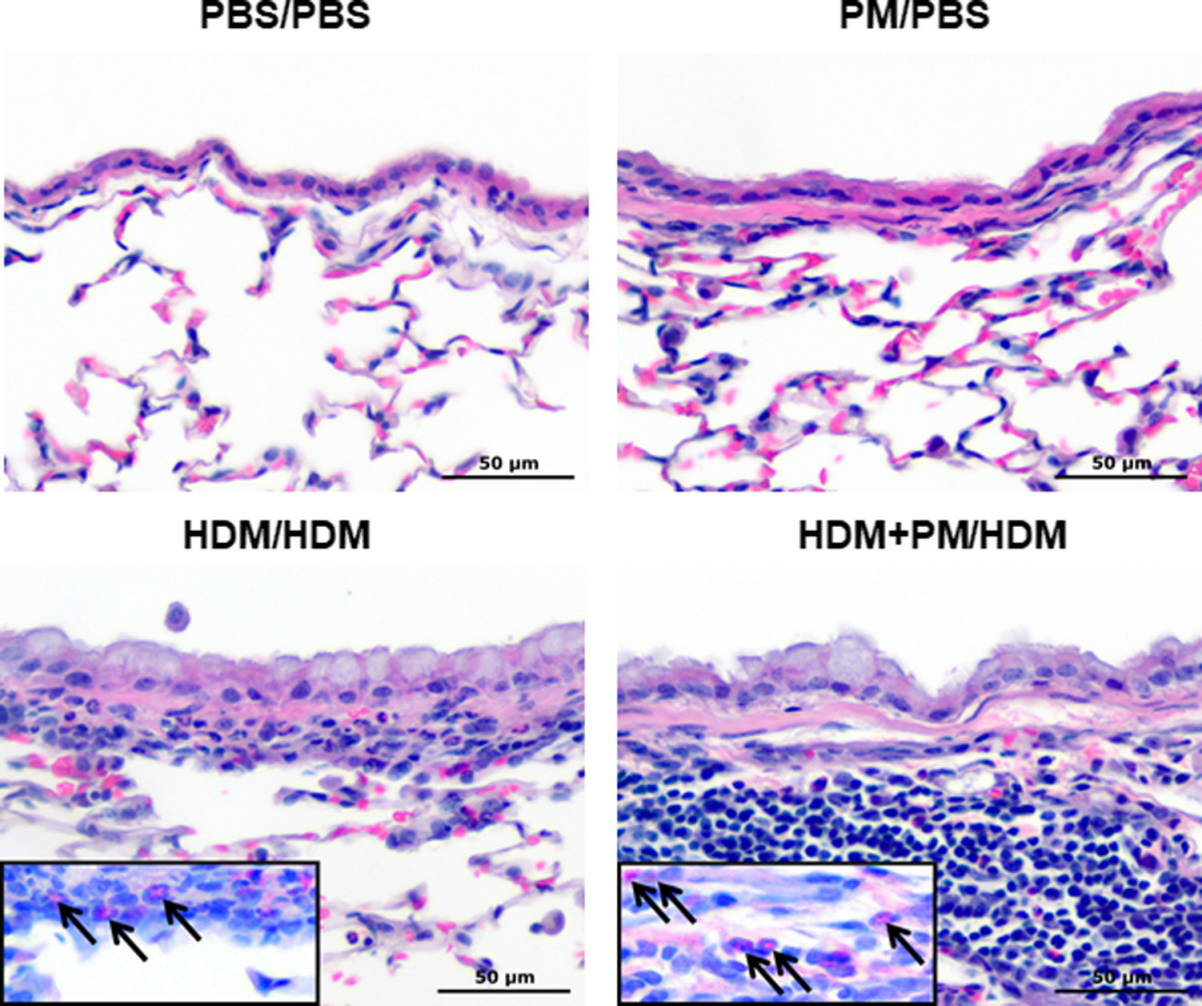
Histopathology of the lung. HDM + PM/HDM treatment induces greater sub‐epithelial influx of monocytes/macrophages and eosinophils compared to HDM‐alone treatment. Micrographs of the epithelium of central airways from paraffin‐embedded lung tissue sections (×400 magnification) stained with hematoxylin & eosin. Inserts show serial sections of lung tissue stained with combined eosinophil and mast cell stain, arrows indicate eosinophils. The scale bar represents a distance of 50 *μ*m.

Histopathology of lung tissue confirmed the migration of monocytes/macrophages, neutrophils, and eosinophils into the pulmonary compartment, with localization to the sub‐epithelium of the central airways of both HDM‐treated groups (Fig. 3). The influx of immune cells beneath the airway epithelium was markedly enhanced in the HDM + PM/HDM group compared to the HDM/HDM group. PM/PBS treatment did not lead to visible inflammatory changes and was comparable to the PBS/PBS control. Both HDM sensitized/challenged groups demonstrated goblet cell metaplasia. The combined eosinophil and mast cell stain (CEM; Fig. 3 inserts) highlights the presence of eosinophils in lung tissue (denoted by arrows).

### Plasma immunoglobulin E

**Figure 4:**
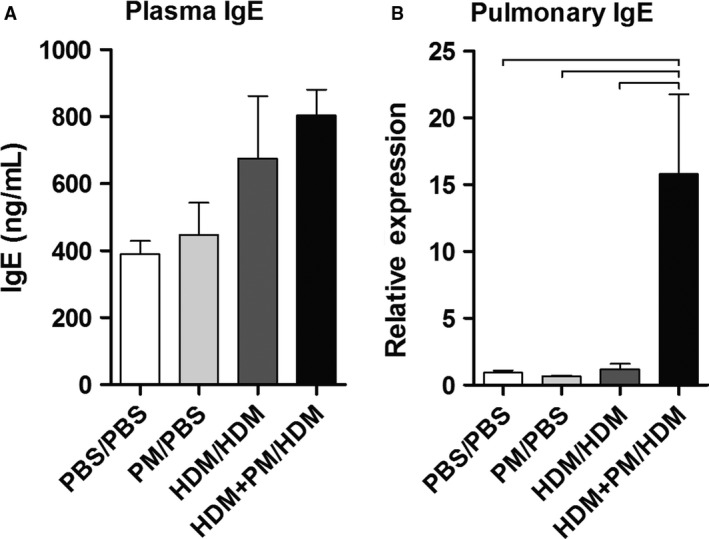
Immunoglobulin E (IgE) protein levels from blood plasma (A) and IgE pulmonary gene expression levels (B). Plasma IgE levels were measured to characterize the systemic allergic response and are expressed as nanograms of IgE per milliliter of plasma (ng/mL). Pulmonary IgE gene expression levels are shown as relative expression to *Gapdh* housekeeping gene. Data are presented as mean ± SEM (*n* = 3–4 mice/group). Plasma IgE protein levels were analyzed in duplicate (A). Bars indicate a significant difference of *P *< 0.05 between groups.

Both a Th2‐mediated immune response and class switching to immunoglobulin E (IgE) antibody production are hallmarks of an allergic response (Gould and Sutton 2008). IgE plasma levels and pulmonary mRNA expression for IgE were assessed to characterize the extent of the allergic immune response (Fig. 4). Both HDM/HDM and HDM + PM/HDM groups show elevated levels of plasma IgE compared to the PBS/PBS and PM/PBS controls, although not statistically significant (Fig. 4A). Pulmonary IgE gene expression, however, was significantly elevated with HDM + PM/HDM treatment compared to HDM/HDM treatment (Fig. 4B). HDM/HDM and HDM + PM/HDM treatments led to a 1.2‐ and 15.8‐fold increase, respectively, in IgE mRNA expression compared to the PBS/PBS control, demonstrating that PM exposure during allergic sensitization significantly enhances IgE gene expression upon subsequent allergen challenge.

### Mucosubstance production and morphometric analysis

**Figure 5:**
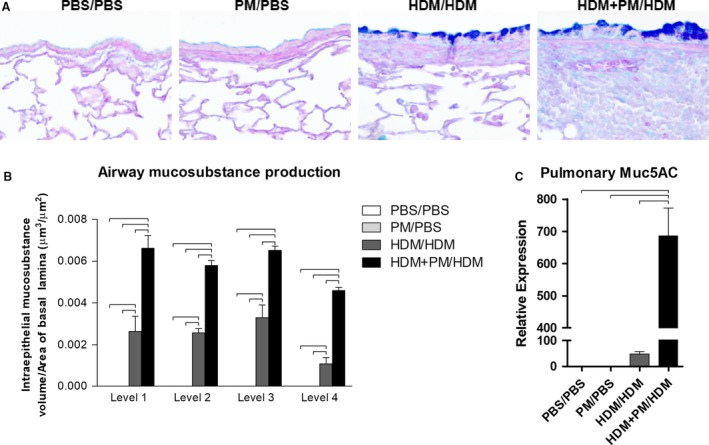
Mucosubstance distribution, abundance, and gene expression. PM exposure during allergen sensitization (HDM + PM/HDM) leads to enhanced mucosubstance secretion upon allergen challenge, compared to allergen‐only sensitized animals (HDM/HDM). (A) Micrographs of paraffin‐embedded lung tissue sections (×400 magnification) stained with Alcian blue and periodic acid‐Schiff (ABPAS). Mucosubstances are stained blue. The scale bar represents a distance of 50 *μ*m. (B) Mucosubstance production was quantified via ImageJ, at four different levels in the lung: Levels 1–4. Mucosubstance production is expressed as intraepithelial mucosubstance volume over the area of basal lamina (*μ*m^3^/*μ*m^2^). (C) Pulmonary *Muc5ac* gene expression was assessed via qPCR. *Muc5ac* gene expression is shown as relative expression to *Gapdh* housekeeping gene. Data are presented as mean ± SEM (*n* = 4/group). Bars indicate a significant difference of *P *< 0.05.

Airway mucus hypersecretion is characteristic of the allergic response (Evans et al. 2009). Morphometric analysis of intraepithelial mucosubstance‐positive cells revealed that HDM + PM/HDM treatment significantly enhanced mucin production at various levels along the main apical‐path airways (level 1) and main axial‐path airways (levels 2–4) compared to HDM/HDM treatment (Fig. 5A and B). These effects were also present at the mRNA level; pulmonary *Muc5ac* gene expression was significantly enhanced by HDM + PM/HDM treatment compared to HDM/HDM treatment (686‐fold increase vs. 47‐fold increase, respectively, Fig. 5C).

### Pulmonary heme oxygenase‐1 expression

**Figure 6:**
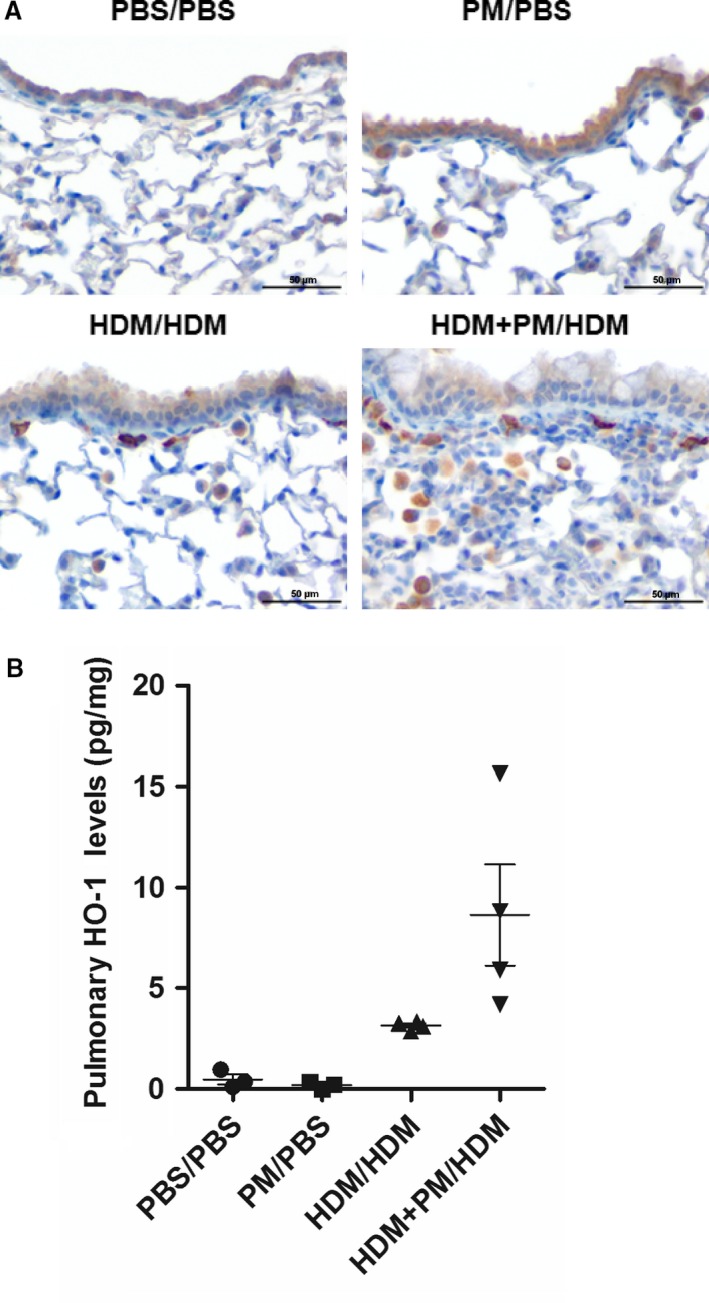
Heme oxygenase‐1 (HO‐1) pulmonary protein levels. (A) Micrographs of paraffin‐embedded lung tissue sections immunohistochemically stained with HO‐1 (400× magnification). The scale bar represents a distance of 50 *μ*m. (B) Pulmonary HO‐1 protein levels measured by ELISA, standardized to total lung protein, and expressed as picograms of protein per milligram of lung tissue (pg/mg). Data are presented as mean ± SEM (*n* = 4 mice/group). Lung HO‐1 protein levels were analyzed in duplicate.

Numerous scientific groups have attributed the inflammatory effects of PM to be associated with enhanced oxidative stress (Brown et al. 2006; Ayres et al. 2008). To explore if PM increased oxidative stress, paraffin‐embedded lung tissue sections were immunohistochemically stained with an antibody against heme oxygenase‐1 (HO‐1), an indicator of oxidative stress. HO‐1 was positively expressed in all treatment groups and was localized to epithelial cells, fibroblasts, and macrophages (Fig. 6A). HO‐1 protein was measured in lung tissue via ELISA; however, comparison of HO‐1 protein levels between HDM/HDM and HDM + PM/HDM groups did not achieve statistical significance (Fig. 6B).

### Pulmonary gene expression analysis

**Figure 7:**
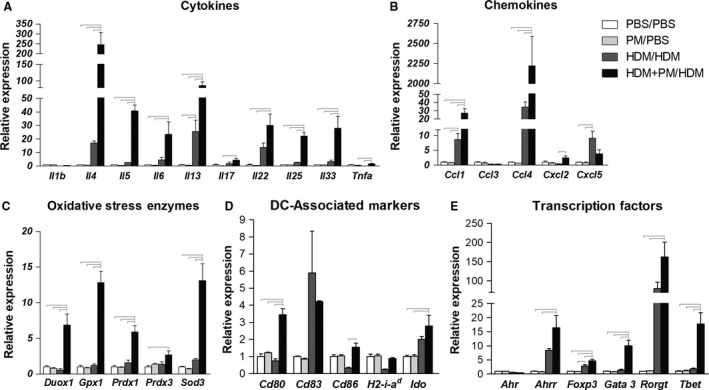
Pulmonary gene expression profiles of cytokines (A), chemokines (B), oxidative stress enzymes (C), dendritic cell (DC)‐associated molecules (D), and transcription factors (E). Gene expression is shown as relative expression to GAPDH or Eef1a1 housekeeping genes. Abbreviations: Il = interleukin, Tnfa = tumor necrosis factor alpha, Ccl = C‐C motif chemokine ligand, Cxcl = C‐X‐C motif chemokine ligand, Duox = dual oxidase, Gpx = glutathione peroxidase, Prdx = peroxiredoxin, Sod = superoxide dismutase, Cd = cluster of differentiation, H2‐a‐i^d^ = major histocompatibility complex II, beta‐chain, Ido = indoleamine 2,3‐dioxygenase, Ahr = aryl hydrocarbon receptor, Ahrr = aryl hydrocarbon receptor repressor, Foxp3 = forkhead box P3, Gata3 = GATA binding protein 3, Rorgt = retinoid*‐*related orphan receptor gamma t, Tbet = T‐box transcription factor, Th1‐associated. Data are presented as mean ± SEM (*n* = 3–4/group). Bars indicate a significant difference of *P *< 0.05 between groups.

The expression of various known immune‐ and allergy‐associated genes in the lung was evaluated (Fig. 7). Gene expression of the Th2 cytokines *Il‐4*,* Il‐5*,* Il‐13*,* Il‐25*, and *Il‐33* were significantly enhanced in the HDM + PM/HDM treatment group versus the HDM/HDM group (Fig. 7A). Moreover, gene expression of the Th2 lineage‐specific master transcription factor *Gata3*, was significantly enhanced in the HDM + PM/HDM group compared to the HDM/HDM group (Fig. 7E). Of importance, expression of *Il‐4*, the cytokine that induces the differentiation of naive helper T cells into Th2 lymphocytes, reached a 246‐fold increase in expression in the HDM + PM/HDM group (relative to PBS/PBS), compared to a 17‐fold increase in the HDM/HDM group (relative to PBS/PBS; Fig. 7A). In addition, gene expression of co‐stimulatory molecules CD80 and CD86, which are necessary for T‐cell activation/differentiation, was significantly raised with HDM + PM/PM treatment compared to HDM/HDM treatment (Fig. 7D). Oxidative stress enzymes *Duox1*,* Gpx1*,* Prdx1*, and *Sod3* were significantly enhanced with PM exposure during allergic sensitization (HDM + PM/HDM) compared to HDM/HDM treatment (Fig. 7C).

With HDM + PM/HDM treatment, certain transcription factors were significantly increased in expression (Fig. 7E). Gene expression of the aryl hydrocarbon receptor (*AhR*) and it repressor (*Ahrr*) were measured as the AhR is a known target of polycyclic aromatic hydrocarbons (PAHs), organic material found in PM. While *Ahr* itself remained unchanged with treatment, expression *Ahrr* was significantly elevated for HDM + PM/HDM compared to the PBS/PBS control and PM control (Fig. 7E). This same pattern was true for *Il‐22* and *Rorgt*, which play a key role in Th17 cell differentiation and *Il‐17* expression. However, *Il‐17* gene expression levels between the HDM/HDM and HDM + PM/HDM groups were not significantly different.

### Pulmonary protein levels

**Figure 8:**
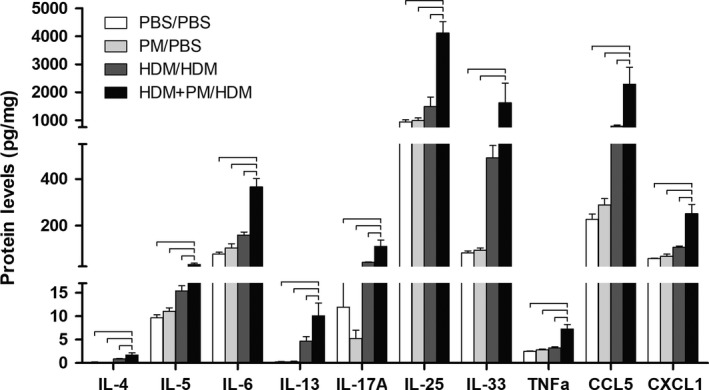
Pulmonary cytokine and chemokine protein levels. Protein was measured by ELISA, standardized to total lung protein, and expressed as picograms of cytokine per milligram of lung tissue (pg/mg). Data are presented as mean ± SEM (*n* = 4/group). Samples were analyzed in duplicate. Bars indicate a significant difference of *P *< 0.05 between groups.

To determine if the observed gene expression changes (Fig. 7) were evident at the protein level, various cytokine and chemokine protein levels were assessed (Fig. 8). The protein expression profiles of IL‐5, IL‐6, IL‐25, and TNFa cytokines showed a similar relationship with gene expression profiles, with the HDM + PM/HDM treatment group demonstrating a significant increase in protein levels of these cytokines compared to the HDM/HDM treatment group. The two cytokines classically associated with Th2‐immune responses, IL‐4 and IL‐13, showed significantly elevations in the HDM/HDM + PM group compared to the HDM/HDM group. In addition, IL‐17A, the primary Th17 cytokine, was significantly elevated in the HDM + PM/HDM treatment group compared to the HDM/HDM treatment group, suggesting PM enhances Th17 immune responses. HDM + PM/HDM treatment significantly enhanced expression of CCL5 versus HDM/HDM treatment, a chemokine known to play a role in promoting T‐cell lymphocyte and eosinophil migration to the lung.

## Discussion

This study explored how exposure to PM_2.5_ during simultaneous allergen sensitization modulates the development of the pulmonary allergic immune response and how these effects manifest themselves upon subsequent allergen challenge. Endpoints of this study included: (1) characterizing the extent of pulmonary inflammation based on recovered BAL cell numbers and cell type; (2) assessing whether the HDM allergen mouse model captured the hallmarks of allergic airway inflammation, including an IgE antibody response, subepithelial inflammation of airways, pulmonary eosinophilia, and mucin production; and (3) defining the expression patterns of various genes and proteins involved in immune and antioxidant responses to gain insight into PM modulation of the allergic immune response.

The results demonstrate that exposure to PM only during allergen sensitization produces long‐term measurable effects upon allergen challenge, leading to enhanced: immune cell migration to the lung, eosinophil and monocyte/macrophage localization to the subepithelium of the airways, pulmonary IgE gene expression, mucosubstance production, and the expression of Th2‐ and Th17‐associated genes and proteins. PM displayed adjuvant‐like effects in enhancing allergic sensitization and inflammation upon allergen challenge, whereas PM exposure alone did not lead to any significant inflammatory effects.

The PM used in this study was collected in downtown Sacramento CA, near three major highways. Chemical analysis determined PM was predominantly composed of organic carbon (49% composition by mass) abundant in PAHs and nonaromatic hydrocarbons. PAH exposure can result in AhR activation (Denison et al. 2002; Denison and Nagy 2003). This is noteworthy given the fact that we found evidence of AhR activation in our model based on induced mRNA expression levels of AhR repressor (AhRR), which is regulated via the AhR signaling pathway (Haarmann‐Stemmann and Abel 2006). Environmental toxins that lead to AhR activation have been shown to regulate the delicate balance between Th17 and T regulatory cell (Treg)‐differentiation in a ligand‐specific fashion (Quintana et al. 2008; Veldhoen et al. 2008). Exposure to PM has also been associated with AhR‐dependent Th17‐immune responses in the lung (van Voorhis et al. 2013; Julliard et al. 2014). Furthermore, studies have shown that diesel exhaust particles can induce Th17‐immunity in the context of allergic inflammation (Brandt et al. 2013, 2015); with some researchers showing that PM exacerbates allergic responses via priming of Th17 immune responses in an AhR‐dependent manner (Castañeda et al. 2018). Our findings are in agreement with such studies, as ROR*γ*T expression, a marker of Th17 responses, and IL‐17A protein levels were also enhanced by PM treatment in the allergen‐treated group. These results support the hypothesis that air pollution exacerbates the allergic immune response by enhancing Th17‐immune responses. However, PAH exposure has also been associated with impaired Treg function via epigenetic modification of the FOXP3 locus in human allergy (Hew et al. 2015). Notably, our model showed a significant elevation in the expression of FoxP3 in mice sensitized to HDM in the presence of PM relative to all other groups (Fig 7E). PAHs may possibly worsen allergic responses by both causing Treg dysfunction while simultaneously enhancing Th17 immune responses.

PM demonstrated potent effects on Th2‐mediated immune responses. The major Th2 cytokines, IL‐4, IL‐5, and IL‐13, were significantly enhanced by HDM + PM/HDM treatment relative to HDM/HDM treatment. IL‐4, the signature cytokine that drives naive T cell differentiation into Th2 lymphocytes, was remarkably enhanced in animals sensitized with HDM + PM versus HDM sensitization (246‐fold increase versus 17‐fold increase, respectively). This translated into a significant increase in expression of GATA3, the Th2 lineage‐specific master regulatory transcription factor, when allergen sensitization occurred in the presence of PM. Elevation of the eosinophil chemoattractive cytokine IL‐5, was apparent in the HDM + PM/HDM group at both the gene and protein level. The elevation of IL‐5, along with CCL5, in this treatment group likely explains the significant rise in eosinophil migration to the pulmonary compartment compared to the HDM/HDM treatment group. Furthermore, significant elevations in IL‐13 gene expression in the HDM + PM/HDM group may explain the enhanced allergic response as IL‐13 plays a pivotal role as a central mediator of allergic inflammation by enhancing IgE production, mucus secretion, eosinophil recruitment, macrophage and dendritic cell activation, as well as airway hyperresponsiveness (Ingram and Kraft 2012). Lastly, expression of the epithelial‐derived cytokines IL‐25 and IL‐33 that promote Th2‐immune responses were also augmented when allergen sensitized mice received PM compared to no PM (HDM/HDM). Collectively, these results demonstrate that PM worsens allergic inflammation in the lung by enhancing Th2‐mediated immune responses.

The modulation of Th2 cells and cytokines by PM during allergen sensitization manifested itself as an enhancement of pathological lung changes that define the hallmarks of allergic airway inflammation. Histopathological assessment of lung tissue from HDM + PM/HDM groups highlighted greater cellular migration of leukocytes, notably eosinophils, to the subepithelial region of the central airways compared to the HDM/HDM group. It is important to emphasize that PM treatment alone (PM/PBS) did not lead to any inflammatory changes at the time of analysis and was comparable to the PBS/PBS control. Both allergen‐treated groups displayed goblet cell metaplasia, epithelial hyperplasia, and a minimal degree of smooth muscle hypertrophy, however, mice treated with HDM + PM/HDM produced significantly higher levels of mucosubstances compared to mice treated with HDM/HDM.

Our next aim was to explore how PM exposure during allergen sensitization modulated the immune system to generate a greater inflammatory response upon allergen challenge. Numerous studies, including our own, have demonstrated that PM exposure leads to the depletion of pulmonary antioxidants initiating oxidative stress mechanisms that both enhance immune cell activation and injure lung cells directly (Brown et al. 2006; Ayres et al. 2008; Li et al. 2009; Carosino et al. 2015). We measured total HO‐1 protein levels in the lung and used immunohistochemical staining to localize HO‐1 expression in lung tissue, as this protein has been shown to be an indicator of oxidative stress (Ayres et al. 2008). Total lung HO‐1 protein levels were augmented in the HDM + PM/HDM group compared to the HDM/HDM group (although not statistically significant) with HO‐1 expression localizing predominately to macrophages. Although PM serves as an exogenous agent of oxidative stress within days of its administration, the observed increase in HO‐1 expression is likely the result of endogenous oxidative stress induced from the degranulation of reactive oxygen species (ROS)‐containing granules from activated immune cells, which are present in higher numbers in the HDM + PM/HDM group. This is evident in the fact that HO‐1 levels were similar between the PM/PBS and PBS/PBS groups at the time of analysis, ruling out long‐term oxidative stress effects. Further support for the hypothesis that immune cells are the source of ROS comes from the observation that recovered macrophages from BAL did not show any engulfed PM, suggesting PM was cleared by day 15. Particle deposition in the lungs triggers alveolar macrophage phagocytic activity within an hour, and typically >90% of particles are engulfed by macrophages within 24 h (Lippmann et al. 1980; Brain et al. 1984). By comparison, PM was administered 10 days prior to the HO‐1 analysis. We conclude that PM‐mediated oxidative stress is an acute response that lasts days. We speculate that the observed increased HO‐1 expression in HDM‐treated animals resulted from greater recruitment and activation of immune cells that release ROS in response to HDM challenge, generating endogenous oxidative stress.

We hypothesized that PM may have other immunomodulatory effects independent of oxidative‐stress driven processes. The PM_2.5_ used for this study was collected in the downtown area of Sacramento, and was rich in organic carbon, including PAHs and hydrocarbons, byproducts of combustion of organic matter such as fossil fuel, which are of increasing concern as a class of AhR agonists. Various studies have demonstrated that traffic‐derived PM near highways worsens allergic symptoms (Peterson and Saxon 1996; McConnell et al. 2006; Kelly and Fussell 2011). To investigate whether PAH and hydrocarbon content in Sacramento PM_2.5_ could be responsible for the observed immunotoxicity and enhanced allergic response, expression of the AhR and its repressor, the AhRR, were assessed since AhR activation can occur via synthetic PAHs and dioxin‐like compounds present in air pollution (Denison et al. 2002; Denison and Nagy 2003). Co‐exposure to PM and HDM allergen induced AhRR gene expression more so than HDM allergen alone, suggesting AhR activation. Mechanistic studies have shown that AhR‐signaling is involved in Muc5AC gene upregulation and mucin production (Wong et al. 2010; Chiba et al. 2011), notably both features in our animal model were significantly elevated in the HDM + PM/HDM treatment group compared the HDM/HDM treatment group.

Various AhR ligands, including PAHs in PM, have recently been shown to promote Th17 polarization and secretion of IL‐17A from T cells in vitro and in vivo via the AhR, in non‐allergic models (Ramirez et al. 2010; van Voorhis et al. 2013; Julliard et al. 2014). How these effects translate to allergic models however, remains unknown. Expression of RORgT, the Th17 linage‐specific master transcription factor and pulmonary IL‐17A protein levels were significantly enhanced by PM treatment. Expression of IL‐22, an additional Th17‐associated cytokine, was also enhanced by PM exposure in allergen treated animals. IL‐22 plays a protective role during early inflammation; however, long‐term secretion and dysregulation of IL‐22 induces tissue remodeling that contributes to chronic inflammation (Eyerich and Eyerich 2015). These observations in our model support the hypothesis that PAH and its components promote AhR activation in DCs during allergen sensitization, bestowing upon these cells improved capability to more potently activate T lymphocytes during immunological imprintation, which manifests as greater Th2‐ and Th17‐immune mediated pathology. Specifically, DC co‐stimulatory molecules (CD80 and CD86) were significantly elevated by HDM + PM/HDM treatment over HDM/HDM treatment indicating enhanced DC activation. Given that PM's oxidative potential does not fully explain the augmented allergic response, these findings may imply that PM is able to modulate the innate immune response through DCs, which in turn amplify the subsequent adaptive immune response.

In summary, we demonstrate that PM exacerbates the pulmonary allergic immune response when exposure occurs during simultaneous allergen sensitization. The enhancement of the allergic immune response by PM is characterized by increased monocyte and eosinophil migration to the subepithelium of central airways, Th2 cytokines, IgE expression, mucosubstance production and, to a degree, Th17‐immune responses. The greater oxidative stress that has been previously observed in allergen sensitized mice that receive PM is likely the result of endogenously produced ROS by immune cells upon allergen challenge. PM‐mediated toxicity may be the result of PAHs directly modulating immunological memory via the AhR in DCs, which translate into augmented allergic inflammatory effects via Th2‐ and Th17‐polarization. Further work is needed to investigate if PM enhances the HDM antigen‐presenting capabilities of DCs and if this translates to enhanced B‐ and T‐cell adaptive responses, as well as the critical role of the AhR in these processes. Identifying the molecular mechanisms through which PM mediates its toxicological effects and enhances immune‐mediated inflammation may shed light on the sharp rise in asthma and allergic disease in the past decades. In conclusion, our study provides evidence that air pollution exacerbates pulmonary allergic inflammation by acting as an immune adjuvant, by enhancing Th2‐ and Th17‐immune responses, and highlights the hazards that environmental components, such as air pollution, pose in promoting asthma and allergy susceptibility.

## Conflict of Interest

The authors do not have competing interests.
